# Practical Remarks on the Discrimination and Appearances of Surgical Disease

**Published:** 1841-07-01

**Authors:** 


### Medico-Chirurgical Review,

### No. LXIX.

**[No. 29 of a Decennial Series.]**

APRIL 1, TO JULY 1, 1841.

Practical Remarks on the Discrimination and Appearances of Surgical Disease; with an Appendix, containing the Descriptive Catalogue of the Author's Collection in Pathological Anatomy; and the Hunterian Oration for 1833. By *John Howship*, Surgeon to Charing Cross Hospital, &c. London: John Churchill, 1840.

This work must be considered as possessing an additional interest and claim upon us, from the demise, since its publication, of the author. Mr. Howship has always evinced great zeal in the cultivation of pathology, and many of his papers have been highly valuable. If we do not owe to him remarkable discoveries, we are his debtors for useful facts, and the volume before us contains, if we mistake not, a considerable share of them.

We do not know how we can better present an epitomized view of its contents than by referring to the introduction. In that, the author tells us, that the first affection adverted to, is a peculiar description of chronic pain in the head; and here discrimination at once confers the power of affording immediate relief. Derangement or disease in the maxillary antrum introduces some interesting illustrations. The next subject is interstitial or ulcerative absorption of the bones of the cranium; and here the very first illustration is calculated to give an instructive lesson, on the necessity for understanding the principles as well as practice of Surgery, that we may proceed with judgment and pursue a right course. The last case adduced under this title is extremely curious, and, in a physiological point of view, very important, on several accounts, especially as exhibiting the operation of a most curious law in the system, that where atrophy of the brain induces a diminution of its volume, the cranium generally follows its contents, by a correspondent change of external form; although M. Cruveilhier, in his admirable work on Pathological Anatomy, gives a very striking exception to the rule. The remarks next in order, regard fracture of the basis-cranii, in which we see the advantage of watching and noting the effects of accident for a series of years; a habit, which now and then furnishes the student with a diagnosis not otherwise to be acquired.

These comments are followed by remarks on specific disease in the cranium, and its contents. Scirrhous tumours in the diploe or in the fibrous tissue of the dura mater. Fungoide tumour in the eye and orbit, or in the dura mater; illustrating the different modes in which disturbance or destruction of the substance of the brain may take place, from absorption, or disorganization. Next come a series of remarks on the phenomena and causes of congestion of the brain, including the powerful influence of mind in its production. Several of the cases may be truly said to possess great practical interest, in reference to the discrimination and treatment of this disease. The consideration of deficient development or subsequent wasting of the limbs, next follows; from affection of brain or of some part of the nervous system, leading on to a number of interesting and various practical illustrations.

The next condition of brain noticed is that of serous effusion; a condition, which may almost always be distinguished during life; and here it is satisfactory to observe, that where the existence of the disease is most clear, and where its progress has induced obvious enlargement of the cranium, a prompt recourse to active treatment may be attended with perfect and permanent recovery.

Injury of the head from the passage of the electric fluid through the brain, comes next in order; and here is drawn a curious and close parallel between the symptoms of concussion, extravasation, inflammation, and effusion, as induced by this cause, and as brought on by the more ordinary modes of external violence. Inflammatory affections of the brain offer some interesting distinctive features; and it may be remarked, that, when the excitement is superficial only, the symptoms may differ essentially, with the tissue principally involved, whether venous or arachnoide. The occasional and curious influence of abscess in the brain, near the origin of the olfactory nerve, in giving rise to a false sensorial impression is noticed, and practically illustrated. The formation of a large abscess in the lateral ventricle, without its having excited the least mental disturbance, is curious; but the production of several large abscesses in the substance of the brain (two of which abscesses are distinctly and demonstrably seen to be situated one within the other) is still more so.

The formation of hydatids in the brain may present symptoms by which this complaint may be distinguished from ordinary serous effusion, which it most resembles. Ramollissement of the brain should excite much close consideration and interest in the practitioner, from the discordance of opinion which still exists as to its cause, and consequently its treatment; while even its diagnostic symptoms are by no means uniformly admitted or generally understood. The practical illustrations under this title may, it is hoped, be regarded as affording some useful and important light.

Effusion of blood within the head is next considered; and whether from an internal or external cause may generally be discriminated. The severely painful affection, tic doloureux, was long supposed to affect only the nerves of the face; but experience has shewn the more extensive range of its influence; and the cases demonstrate its direct power of deranging not only the nerves of sensation in the body and limbs, but the various ganglia and organs supplied by the sympathetic system, the nerves of sense, and even the sensorium itself. The discrimination of the causes of compression of the spinal cord in the neck is important, and the agency of some of these causes obscure; several of the illustrative remarks on their operation will be found interesting and rare.

Tumour of the thyroide gland is not subject to much variety; it is occasionally liable to common inflammation and abscess, but very rarely indeed to scirrhus. The extent of infiltration into the cellular tissue, in the first case of bronchocele, is very extraordinary.

Chronic ulceration of the epiglottis, or larynx, easily distinguished, but nor easily relieved, is in fact one of the most distressing and embarrassing cases that can occur ; the misery induced by this disease can be understood only by referring to the annexed illustrations. The remarks on scrofulous disease in the cervical vertebræ may serve to illustrate, in some degree, the symptoms and appearances. Malignant disease of the face, or of the tongue, is noticed principally to establish its diagnosis. Spasmodic stricture in the œsophagus furnishes several interesting practical remarks and illustrations : as simple spasm, as inflammatory spasm, and as occasionally incurring a state of stomach no less serious than the complaint itself. The observations on malignant disease in the same part may much assist in the discrimination of these complaints. Abscess in the œsophagus is shewn sometimes to prove fatal, but the illustrations also demonstrate that it may terminate favourably.

Affections of the heart and circulation next follow. Adhesion between the heart and pericardium ; enlargement or hypertrophy ; aneurism ; rupture of the heart ; are each practically illustrated. Of the formation of polypus in the heart, independent of the comments, two cases are given, which appear to prove most clearly, not only that these concretions may form during life, but occasionally cause death. On disease in the valves of the heart, the illustrative remarks and appearances may, it is hoped, be regarded with interest and advantage ; urging the importance of the stethoscope, demonstrating the fatal result of the disease, and pointing out the means by which its early progress may be detected and checked.

Aneurism of the thoracic aorta presents an important subject for study, with a view to its discrimination ; the practical illustrations here adduced are calculated to throw some interesting light on the symptoms, appearances, and treatment. Abdominal aneurism also offers some valuable practical illustrations ; in three of the cases aneurism was cured, in one by an operation, in the two others by the natural process ; one case is added, not of aneurism, but of disease simulating aneurism so closely as to defy all power of discrimination. The consequences of tumour formed in the coats of the aorta, near its origin, are shewn in two curious and interesting cases. Where the course of the circulation is interrupted by obliteration or obstruction of the aorta, the facility and efficiency with which the collateral circulation may be established, is most admirable and astonishing. Next follow remarks on obstruction from varicose enlargement, or from inflammation, of the veins ; the discrimination of these complaints being assisted by a variety of practical illustrations. Obstruction to the circulation from local disease of viscera, gives rise to much interesting illustration of various kinds. The first case mentioned of soft white tubercles surrounding the large vessels of the heart, is believed to be of rare occurrence.

The remarks on the symptoms and discrimination of inflammation in the lungs, and its consequences, in various forms of effusion, may, it is hoped, be found useful at the bed side ; they are illustrated by examples of hydrothorax, empyema, abscess, or ulceration with hæmorrhage, hæmorrhage without ulceration, and, lastly, tubercular disease. Then come the symptoms and discrimination of disease in the liver, commencing with inflammation, acute and chronic, abscess, purulent hydatid cyst, and serous hydatid cyst. Irritation from biliary calculi introduces several exceedingly curious and interesting practical illustrations; among which may be mentioned the enormously enlarged ducts within the liver, in the absence of a gall-bladder; the detection of permanent spasm of the gall-ducts; the escape of calculi by ulceration; and the large fungoid tumor, with a collection of calculi in its centre.

These are followed by affections of stomach, spasm, inflammation, effusion, ulceration, mortification, hæmorrhage, and organic or specific disease. Then succeed the diseases of the intestinal canal; altered action of the mucous exhalants, separating albumen, pus, or blood; deranged muscular action or spasm, with the means for more efficiently detecting its existence and precise seat, noticing certain very peculiar symptoms, probably the result of reflex irritation, obstruction, and intus-susception, inflammation and ulceration of the mucous tissue, and occasional influence of ulceration on the nerves of the lower extremities, abdominal abscess, and specific disease in the bowels.

In the last place are described the various forms and appearances of hernia, some of the illustrations of which are believed to be very rare; the case of thyroideal hernia, especially, presenting a diagnostic symptom that appears not to have been before observed.

In a work made up of miscellaneous facts, like the present, a systematic analysis would be impossible. All that can be done is to select those observations that are likely to be useful or are novel, and lay them before our readers. The genius of medical literature in this country, and the practical tastes of the profession are not altogether repugnant to works of this description.

### ON CHRONIC PAIN IN THE HEAD RELIEVED BY INCISION.

There are, says Mr. Howship, certain cases of chronic pain in the head, occasional or constant, from injury or otherwise, generally seated in one spot, over which the scalp is painful, tender, and somewhat tumid; in which medicine is of little or no use. In these cases, a division of the scalp, by an incision, carried across the spot down to the bone, will prove the most efficient treatment.

The relief experienced from this operation is such as can only be explained in the light afforded by pathological anatomy, which enables us to see that, in many chronic affections of the scalp, the membranes of the brain, and especially the dura mater, participate; inducing pain, and all the ill consequences of congestion of the brain.

Mr. Howship relates two cases. The first was that of a lady aged 31, who had suffered from pain in the head, for years, in spite of all medical treatment. Division of the tumid and tender scalp gave immediate and permanent relief.

The *second case* is still more interesting. H. D., a coachman, a sober man, complained of severe pain in the head, distressing him many years, and of late so severe that he was scarcely able to sit on the box; and when up, dreaded the attempt to get down. Medicine useless; nearly distracted, and tired of his life. He said that, sixteen years before, he struck his head, by a fall, upon some soft meadow ground; that from the confusion that followed he soon recovered, but became subject to pains in the head, especially in the seat of the injury, over the left parietal bone, where the scalp was still sore, painful, and puffed. Mr. Howship cut through the scalp and thickened pericranium, down to the bone, which was uneven and rough, but not carious. The night before this operation he had not slept five minutes, from severe pain, but said he felt relief the moment the bone was exposed, and the following night rested better than for months before, getting three or four hours' refreshing sleep.

The wound nearly healed in a month; the old pain partially returned, with a sense of numbness in the side of the face, as before the operation; but the wound again suppurating, remained open several months longer, and then healed permanently. From this time he lost all his complaints; continued well; and some years afterward told Mr. H. he never was in better health than he then enjoyed.

### On Ulcerative or Interstitial Absorption of the Cranium, from Irritation or Injury.

Mr. Howship was consulted by Mr. B., a gentleman who stated that, thirty-two years previously, he had suffered severely under the removal of nearly the whole necrosed right, and part of the left, parietal bones, from syphilis. He recovered his health, and for very many years remained well, with a large depressed cicatrix, beneath which was a smooth expansion of new bone, deposited in the granulations from the dura mater.

Lately, he had been rendered extremely uneasy, by a part of the cicatrix spontaneously ulcerating, without local pain or other sign of ill health. The ulceration extending, a few thin, small scales of new formed bone came away. Within a fortnight he was attacked, for the first time, with gout. The ulceration healed, although his gradual recovery occupied several months; but there was no subsequent return.

Mr. Howship conceives that it was the irritation of the latent gout that gave rise to the inflammation. We are not so sure of this. Disease of the bones, after syphilis (probably mercurial), is very apt to recur without any ostensible cause, and *it* might as well give rise to the gout, as the gout to it.

"Occasionally," says Mr. H., "from external injury, in young children, some extent of the skull has been removed by interstitial absorption. If blood be effused beneath the scalp, leeching may lessen its quantity: but if not, should it be let out—and when? An early opening may add a second irritation to the first; and any delay in letting it escape may perhaps operate to induce absorption of the subjacent bone. As a rule, I think it best to make an early opening." 7.

Mr. H. relates two cases that occurred in children. We shall introduce one.

*Case.*—T. G., aged three years, fell down an area, struck the back of his head, and was taken up senseless. Under proper treatment he recovered; but a month after, a considerable tumour remained just above the lambdoidal, and over the sagittal suture. The elevated scalp soft and yielding; the pulsation of the brain was distinctly perceived in the tumor, even by the eye; so that any opening incautiously made through the scalp might have injured the dura mater or brain, and destroyed life. The opening in the skull was three inches long and one broad; the ossific margin in parts presenting a raised and compact, or slightly tuberculated edge.

Neither is this liability to absorption from external injury confined to an early period of life. In one instance, Mr. H. detected a small opening produced in the vertex of the skull, through which the brain pulsated, in an aged woman; the consequence, she said, of a bruise from a blunt iron, many months before; yet productive of little pain or inconvenience.

Mr. Howship mentions the particulars of a curious

*Case of Extensive Absorption and Necrosis of the Cranium, with Atrophy of the Brain.*

R. H., aged 36, who stated that she had never had syphilis nor used mercury, had suffered for the last fourteen years from extensive necrosis of both parietal bones; the separation of numerous pieces from both jaws, with or without teeth; portions of the left frontal bone; the angle of the right lower jaw; and more recently the mastoide process of the right temporal bone, which came away by the mouth, while the attachment of the sterno-mastoide muscle nevertheless remained undisturbed.

On the superior part of the right side of the head, the brain appeared to have been adequately defended by a formation of new bone beneath the extensive cicatrix upon the scalp, where pulsation, formerly obvious, could not now be felt.

The most curious point, however, in this case is, that although the deficiency of bone in the cranium is partially supplied, the form of the skull has undergone an astonishing though slowly progressive change. The right side of the cranium has sunk to a much lower level than the left, the corresponding hemisphere of the brain having sunk also, in consequence of progressive atrophy of its substance; for the configuration of the head demonstrates that in this direction the cerebrum must have undergone absorption of a part of its mass, nearly or quite equal to one pound weight. It is remarkable, also, that in this case the deficiency in the cerebrum being principally on the right side, the defective condition of the nervous and muscular powers of the limbs is on the right side also.

The superior part of the frontal region has at the same time sunk extensively on both sides, completely changed the aspect of the profile. The mental operations are remarkably disturbed. The only medicine that has been of any service in relieving the extreme feelings of irritation that exist about the head is sarsaparilla.

"This extremely interesting and curious change in the frontal bone, I mentioned to a gentleman well known for his distinguished labours in phrenological science. I supposed it new; but he immediately pointed to a cast demonstrative of a similar change in a late illustrious personage, who, in the decline of intellectual power, sustained a great diminution in the volume of the anterior lobes cerebri; the sinking of the cranium, insensibly following the declension beneath it, from absorption of the brain; as proved by the casts taken."

*Fracture of the Basis Cranii not fatal.*

There can be no question that fatal as this accident usually is, it occasionally does not prove so. Mr. Howship relates two interesting cases of recovery. One we shall introduce.

*Case.*—T. C., 68, fell down stairs, upon his head. Blood flowed freely from the mouth and ears, through the night; the discharge from the ears subsequently becoming purulent. Symptoms of concussion were soon relieved.

Six months after, with much pain at the back of the head, he could obtain sleep only with the back of his head on a pillow, but not on either side; and was often obliged to sit up in bed all night, holding his head with his hands. Occasionally he was delirious and rambling, but never violent, with severe pain in the night, always less in the day.

Two years after the accident, this man still rested very badly; subject to stupor and giddiness, even in the day, and often starting suddenly up in the night, as if delirious, when not so. One night he jumped up in bed, and began talking strangely; his wife accused him of being light-headed, but he said no, he was perfectly awake, and sensible.

"It appears to me, then, that where a patient, under injury of the basis of the cranium, may have partially recovered; by the presence of the symptoms just noticed; bleeding and suppuration, in one or both auditory passages; congestive and uneasy feelings, about the basis of the brain; want of power to bear pressure on the head, and the mild nocturnal delirium; the practitioner may be instructed to give a guarded and cautious opinion, as to any eventual restoration to perfect health." 15.

Temporary Amaurosis, from Affection of the Optic Nerve.

It would appear probable, says Mr. H., that a temporary amaurotic blindness may, now and then, arise from a transitory suspension of power in the optic nerve, in which, from the undisturbed state of the organ of vision, on the one hand, and the absence of any sign of congestion or affection of the sensorium on the other, we seem obliged to admit that the defect must be confined to the optic nerve; the temporary character of the attack at the same time forbidding the inference of existing organic disease.

*Case.*—Mr. F., aged 30, mentioned to Mr. Howship a curious affection of sight to which he was liable, about six years before. He had a feeling of numbness in the right eye, and, within a minute, total loss of sight, so that he could not perceive the flame of a candle when held to it. This lasted three or four minutes, and then gradually subsided; at first he would see the light without distinguishing objects, and in ten or fifteen minutes was quite well. This attack, which returned twenty or thirty times in the space of six months, was never attended, preceded, or followed by pain or other complaint in the head. He had requested a medical friend, more than once, to look at the eye during the attack and compare it with the other, but he could discern no difference between the two.

### ON DEFICIENT DEVELOPMENT OF PARTS.

Mr. Howship alludes to a series of cases having one character in common, that of deficient growth or development, or a wasting of the muscular structure of the limbs, in consequence of affection of the brain from which the nerves proceed, or of disease in the vicinity or course of these nerves.

These complaints are all essentially characterized by an inadequate transmission or unequal distribution of nervous influence.

Mr. Howship relates two cases of paralysis and deficient development of the limbs, arising from the cerebral congestion produced by teething. We quote one.

*Case.* E. F., a healthy child, eighteen months old, from the irritation of dentition, suddenly lost the use of the left side; she was no longer able to stand on the leg or move the arm, which, if raised, fell powerless. Mr. H. directed a blister to be kept open on the neck, and the bowels to be cleared. In a week she was able to stand a little; the improvement more slow in the arm, the fingers could only be partially moved in a month. In three months apparently recovered; in six months the blister was allowed to be healed.

It was curious to observe, during subsequent growth, the deficient symmetry in the two sides. At thirteen years, the left arm was much smaller, and one inch and a quarter shorter than the right; a similar deficiency also existing in the leg. At eighteen, the left arm measured one inch and a half less in length than the right; and the left leg, only two-thirds the circumference of the right, was two inches shorter than the other. She was still unable perfectly to extend the fingers of the left hand, but could walk firmly and freely.

Mr. H. also gives a case of deficient development of the right arm and leg from congestion of the brain, produced by fever. From these he passes to cases of another sort.

1. *Case of deficient Development from External Injury.*—E. T. 14. Fell down stairs when four years old, and bruised the hip. She subsequently had repeated abscesses about the hip-joint, followed by slow retraction of the limb. The retraction eventually subsided, but the deficiency in growth remained. Mr. H. found, on examination, the hip-joint sound, but the affected leg much emaciated, very weak, and, when measured, four inches shorter than the other.

2. *Case of deficient Growth, from Cold, by sitting on Damp Grass.*—C. C., æt. 20, when two years old, was carelessly laid on the left side upon some damp grass. She took cold, her left leg became weak, and some time after was observed not to keep pace with the other in growth, becoming thinner, smaller, softer, and shorter. On comparing the limbs together, Mr. H. found the left leg and thigh very much smaller and weaker, and three inches shorter than the right; and as to power, she was not able to walk more than a quarter of a mile a day.

"In several cases of chronic dysentery, I have seen lameness and wasting of the lower limbs; in one particularly, in a young man, the patient became perfectly and permanently helpless, the thighs drawn up, the legs completely retracted and incapable of motion. In these affections the diagnosis will be sufficiently easy: the absence of any organic disease in the limbs and the presence of symptoms demonstrative of dysenteric irritation in the bowels, cannot fail to explain the character of this complaint." 32.

Mr. Howship adds, that the mere act of volition, unequally exerted, the mere habit of using one limb more freely or frequently than the other, may lead to the same result; as in the following case.

*Case.*—J. C. a strong, healthy man, applied for a high-heeled shoe, his left leg being shorter than the right. He said he was very well till bound apprentice to a turner, he found that standing constantly on the left leg, and working the treadle as constantly with the right, pain often distressed his left knee, and the limb gradually declined in growth.

On comparing the limbs, Mr. H. found much enlargement about the left trochanter, apparent shortening of the neck of the femur, and a degree of stiffness, at first appearing to arise from anchylosis. The left, just three inches shorter than the right leg, was extremely thin and deficient compared with the right, which was muscular, large, and vigorous.

### Effusion of Serum within the Head.

Mr. Howship remarks, what we confess gives rise to some misgivings in our mind, that—"There is an occasional symptom especially diagnostic of external hydrocephalus or effusion between the membranes; when present, I should say it is impossible to mistake the seat of the deposit. One of the cases in which I observed this symptom most distinctly was the following."

Mrs. D., 53, with hydrothorax and anasarca, was for years subject to occasional attacks in the head, in which, always disposed to doze, her senses were observed by all her family to be yet awake to the lightest and least impression; but the peculiar symptom was this, that, whether in the night or day, she would be at one and the same time light headed, yet perfectly sensible; speaking to herself incoherently, yet, if spoken to, answering clearly and rationally. On examination, Mr. H. found abundant serous effusion between the membranes, but no accumulation in the ventricles.

### *Dryness of the Pia Mater.*

Mr. Howship has never witnessed but one case of this.

*Case.*—J. S., 37, of sober and silent temper, complained of violent pain in the head and loins; the latter arose from red gravel, which was occasionally passed with the urine. The violence of head-ache made him stagger and affected his sight, yet he could get no sleep. In dressing his horses, it was often expected he would get kicked, for he frequently went to the heels instead of the head, not seeming to know what he was about. Mr. S. visited him before his death, to draw off his urine; the eyes insensible to light, the pupils dilated, and the mental and physical powers entirely oppressed. The next day he died. On laying aside the dura mater, Mr. H. found the arachnoide and pia mater unusually dry, just as if exposed for twenty-four hours in a warm air, so dry as to feel positively firm and crisp. The ventricles contained about four ounces of serum; the medullary substance of the brain was so firm, that the cavities did not even partially collapse on the escape of their contents.

### ON INJURY BY LIGHTNING.

"In my Course of Lectures on Surgery, I am in the habit of comparing concussion of the brain from external violence with the impression induced by the electric shock; but, on following out the parallel, it becomes extremely doubtful whether concussion should be selected in preference, when so many widely different results also arise from this peculiar description of injury. It may be supposed scarcely necessary to devote much pains to ascertain the cause when so active an agent has been concerned, for these phenomena loudly proclaim themselves; and if instant death was the invariable result in every such case, it might be an inquiry of comparatively little importance; but when we find that all the various effects of injury from other causes are occasionally produced by lightning, we see the evident and high importance of considering and discriminating as correctly as possible, not so much the cause, as the precise changes of condition to which it may have given rise, in order that we may thence be enabled to decide with advantage upon the most appropriate treatment." 41.

Mr. Howship adds that, as the injury sustained depends not on the quantity but the intensity of the electric fluid, the influence of one and the same shock will occasionally induce strange diversities of effect, according to the position of individuals or the conducting power of substances near them at the moment of the accident.

One of the most remarkable circumstances regarding the effects of the electric shock on the living body is this; that while the mildest power incurs only a slight, sudden, contraction in the muscles, with sense of pain in the nerves, ligaments, and joints, and a more severe shock excites violent convulsive action, the most severe shock, demonstrating the greatest intensity of power, is productive of effects of a diametrically opposite character. The rapidity of thought is proverbial, and the quickness with which the convulsion responds to the passage of the ordinary electric shock, is sufficiently remarkable, but under the greatest intensity of power, the passage of the electric fluid seems too quick even for the nerves themselves; for instead of the body being most violently disturbed, it is not moved at all; and if the person was sitting by the fire, or asleep, he so remains to all appearance, until discovered to be dead.

Where sudden death is thus produced, it appears that the transmission of the shock through the body at once exhausts the vital power, and completely destroys the living principle ; and this takes place with such inconceivable rapidity as to effect its purpose before even the nervous system can take the alarm ; and that the living principle is instantly destroyed, in the solids and fluids, is evinced by the muscles not stiffening, the blood not coagulating, and the whole body sinking almost immediately into putrefactive decomposition.

Mr. H. relates several cases—one of slight injury from lightning, which merely produced red marks in the skin of the back, of a delicate arborescent character, not painful or elevated, but slightly heated—another case, in which a stroke of lightning gave rise to all the symptoms of concussion of the brain—a third case, in which, after a lightning stroke, concussion, followed by violent tremors and febrile excitement, terminated in purulent effusion between the membranes of the brain—and finally, several other cases in which the effects were such as would ensue from rupture of a blood-vessel in the cranium.

Mr. Howship relates some interesting cases of *Abscess in the Brain,* without, however, we are afraid, rendering our diagnosis much more certain. Speaking of *Hydatids in the Brain,* he observes (we wish we could be so confident):—The distinguishing symptom here appears to be an occasional attack of convulsion or insensibility, which scarcely disturbs, in the least degree, the intellectual functions; from which attack, where the case presents in its most simple form, the patient is absolutely restored to his activity of body and clearness of mind almost in an instant, the attack coming on and going off suddenly.

### On Ramollissement of the Brain.

Mr. Howship makes some observations on the causes and on the treatment of this serious affection. After noticing the inflammatory hypothesis of Lallemand, and the opinion of Rostan, that it depends on a morbid state of the cerebral vessels, he remarks :—

"My own opinion is with M. Rostan, that ramollissement may arise as an accidental consequence of inflammation, yet that it certainly does often occur without any evidence of preceding inflammatory excitement. But I also believe that it may take place without inflammation on the one hand, or disease of blood-vessel on the other. The importance of this opinion, if correct, must be at once admitted, seeing that it presumes the disease to be, in certain cases, so far more within the reach of relief, than was before supposed. I feel assured it may arise in this way, and that in two cases to be presently related, it did so arise, from excessive mental fatigue, inducing long continued congestion of the brain; and in one of these cases it will especially be seen, that the adoption of an antiphlogistic course would soon have destroyed life, but that a watchful attention to the indications that presented, and the persevering direction of the most powerful tonics and anodynes, proved the obviously efficient means of saving and restoring the patient." 58.

Mr. Howship criticises the sentiments of M. Rostan on the incurability, or something very like it, of ramollissement, and then presents us with an account of the symptoms as they have occurred under his own observation.

"We now then come to the symptoms, by which ramollissement may be discriminated. As I have seen them, they may be said to include the following. Occasional pain, uneasy feelings or giddiness in the head, the impressions through the senses disturbed, the least sound insupportably agonizing, moderate light distressing, the feelings now and then so acute, that the lightest touch extorts most piteous cries, the sentient nerves in the extremities deranged, with feelings of numbness, or of a sandy sensation about the toes and fingers, stiffness in the limbs, rendering the walk unsteady or unsafe, the temper of mind changed to extreme impatience, uncontrollable dejection, or absolute indifference, total want of sleep, or, when dozing, disturbed by most frightful dreams, the mental faculties invariably affected, perception slow, judgment difficult, memory feeble or false, imagination gone, ideas confused, the patient dreads and perhaps cannot endure the least conversation, or if for a few minutes exposed to it, subsequently sinks into the most alarming state of exhaustion. As the disease advances, the patient, especially under the least mental occupation or excitement, is liable to a sudden attack, something between coma and convulsion, in which, although deprived of utterance, and to appearance near death, he is perfectly conscious of his state. From this attack he revives, and after an uncertain interval sustains a return, more severe than before, perhaps with paralytic symptoms, under which he at length sinks.

These then are the leading symptoms. They rarely all occur in the same individual, but the presence of any two or three of them would be sufficient to enable the attentive practitioner to discriminate this most fearful disease. If, for example, the sensations described in the head occurred in connection with the morbid acuteness of sensation in the skin or in the ear, together with any of the other symptoms, they would offer a combination, which, regarding its steady permanence, could scarcely be supposed to be the consequence of inflammation, hysteria, or any disease we yet know of, except that now under consideration." 60.

Mr. Howship relates several cases of fatal ramollissement, and two instances of cure. We shall introduce the shorter of the two, and leave it to our readers to decide what degree of credit they will attach to the belief that the affection was really cerebral softening.

*Case.*—Mr. F. 50, a gentleman in the law, long subject to uneasy feelings in the head, had to visit Chester. Anxious occupation through the preceding week, in considering and arranging the various causes for trial, had made him much worse; and when in court, in the midst of his duties he fainted outright, sunk down completely powerless, and slightly convulsed, yet conscious, hearing all that was said, although unable to speak. Dr. Thackery saw him, and directed aperients, spare diet, and quietude. A relative went down, brought him to London, and Mr. Howship found him complaining of frequent returns of violent shootings through the right temple, and faintings; also a sense of numbness all through the right side, with sandy feelings in the hands and feet. Dr. Hooper visited him with Mr. Howship; his bowels were kept freely cleared, which, with an occasional gentle opiate at night, light diet, and the most perfect quietude, were beneficial. The fainting fits and feelings of exhaustion became less frequent and less severe. The pulse, never excited, was now soft and small, at sixty. The strength and power in the limbs, in the course of a month, much improved; and the preceding distortion of the features and closing of the left eyelid, had quite disappeared. He still walked with a stiff and awkward gait, the soles of his feet not seeming to himself to feel the floor; and so weak, that one morning he fainted on the night-chair, and being alone, fell to the ground.

As to the previous affection of sight, he told Mr. H. first came the double sight, then the sickness without vomiting, and then the fainting in court. The double sight was not strictly so, but a multiplying of one object to many. Or, if he took a pen and put it down on the paper, it was placed half an inch away from where he intended; and when he had written, he found it was in the wrong place. He observed there was the same error in kind now, only much diminished in degree. There was also a little flickering in his sight still; but otherwise he felt quite as well, and even better now, than before he left town.

For many subsequent weeks, this gentleman was directed the vinum ferri, the shower bath, a nutritious diet, and wine; the bowels being constantly attended to. Under this plan, he so far recovered as entirely to lose all his complaints, except that he had still a very slight sense of occasional sandiness in his hands, a peculiarity in feeling which remained so late as six years subsequent to the attack.

We certainly know of no positive reason why ramollissement of the brain should not in its earlier stages be curable. The difficulty is to say when the affection really exists, or, rather, when *such* a morbid lesion *has been* cured.

We pass over several subjects, to pause at two, not very common cases, one of inflammation, the other of abscess of the thyroid gland.

Case.—*Inflammation in the Thyroid Gland.*—J. D., 16, took a severe cold, the submaxillary glands becoming somewhat, and the left lobe of the thyroid gland more, enlarged. Mr. H. found the latter swelling firm and hard; the skin covering it dry and hot. The tumor, closely attached to the base of the larynx, moved with it in deglutition. For a day or two, with heated skin, quick pulse, and white tongue, stinging and shooting pains were severe. A grain of calomel, ter die, in a few days subdued the symptoms, and he was relieved from all uneasiness; the indurated and horny feel of the swelling subsequently, but slowly, subsiding.

Case.—*Inflammation and Abscess in the Thyroid Gland.*—A mail guard, exposed to a deep snow, took cold, felt poorly and stiff about the jaws; inflammation settling with much heat and tumor about the larynx and trachea, impeding respiration to an alarming degree. The tumor, heated and red, was firm as horn, with feverish disturbance. Although the larynx was nearly fixed, it was evidently enough inflammation of the thyroid gland. Poultices and fomentations, assisted by tonics, in a few weeks induced suppuration, and the abscess broke and discharged several ounces of pus, to his relief and comfort. He now breathed more freely, and rapidly improved in appetite, spirits, and strength; the abscess healing in about three weeks.

### Suppuration in the Sub-Mucous Cellular Tissue of the Larynx.

We notice the following case for reasons that we shall state immediately.

*Case.*—A. B., 30, had a succession of acutely painful, and actively suppurating boils, on the thigh, forearm, and back of the hand. When the ulcer on the forearm, which had discharged portions of fascia and tendon, had been open nine or ten days, the secretion of pus suddenly diminished, and in two days ceased entirely; and on the same day he felt uneasiness in his throat. In a few days, respiration became impeded, from some cause, situated evidently in the larynx. Towards evening, the difficulty in breathing was alarming, his features betraying most extreme anxiety and distress. Supported in bed, a consultation was immediately called; antispasmodics directed, and a blister laid on the chest; at six A. M. he expired.

*Post mortem.*—The cause of death was confined to the thyroide catilage, the space within which had been so restricted as to produce suffocation. The cartilage divided, the cellular tissue connecting the mucous membrane to the cartilage was inflamed, thickened, and diseased, especially just beneath the arytenoide cartilages, where it formed a spongy, elastic cushion, projecting from each side, and closing the passage into the trachea. This spongy substance, cut into, numerous globules of a thick purulent matter started forth; the colour and consistence of the pus, and other circumstances of this case, presenting a strong analogy to the character of carbuncle.

We draw particular attention to this case, because it is the representative of a class. It is, unfortunately, far from uncommon for a patient to complain of what seems and is treated as a simple sore-throat, and suddenly to be cut off. On examination after death, suppuration is discovered in the sub-mucous cellular tissue of the larynx. It is hard to lay down any diagnostic signs of this affection. But when the symptoms seem severe beyond their apparent cause, and the expression of distress is disproportioned to it, we should recollect the possibility of suppuration and beware.

### STRICTURE OF THE ŒSOPHAGUS.

"In the detection and treatment, the superiority of the silver ball and elastic curved wire, over the ordinary instrument, the bougie, cannot be too much insisted on, exciting much less irritation, and conveying much more information, as to the state of the pharynx, the precise extent of the contraction, and exact condition of the œsophagus below." 93.

The following case is not devoid of interest:—

CASE.—*Inflammatory Spasmodic Stricture of the Œsophagus.*—"Mrs. E., 63, from severe cold and sore throat, had great and increasing difficulty in swallowing. Afraid of losing a desirable situation, she concealed her complaint, and foolishly neglected her health, until, from want of support, she became emaciated, typhoide, and so weak that she could not stand. In this state I found her, with black and parched tongue, small rapid pulse, hot skin, and thirst. With difficulty I persuaded her to let me introduce the smallest silver ball to a contraction at the bottom of the pharynx, which the instrument would not pass. Unable to take medicine, incapable of taking food, and evidently perishing from want, she was nevertheless sufficiently obstinate to throw away still more valuable time, in resisting for several days her daughter's entreaty and my persuasion, to allow the instrument to be again introduced. She then did submit, and I soon passed the stricture, so that now she could get down small morsels of meat. But now another difficulty arose, for although the food passed fairly down, it was almost directly rejected again. It appeared that the stomach, so long inactive, had become so irritable and contracted, as to be scarcely capable of relaxation; a difficulty in which medicine appeared likely to be of little service. She was therefore enjoined to persevere in taking nourishment, solid or fluid, in small quantities, frequently repeated, notwithstanding its rejection. This she did, and soon began, very gradually to improve. The feverish excitement slowly subsided, the power of retention returned, the strength was restored, and she perfectly recovered." 96.

We are induced to believe that patients are occasionally treated for stricture of the œsophagus, as for stricture elsewhere, without any just occasion. Nor is the mistake always a harmless one. Bougies in the hands of those unaccustomed to them are sometimes dangerous weapons, nay, they may be so even in good hands. Sir A. Cooper used to relate a case which occurred to Sir E. Home and himself. He had found a difficulty in passing an instrument down the œsophagus; Sir E. Home was called in. He took the instrument, passed it in good style, and injected through it milk and water into the *stomach.* Violent symptoms soon set in, and in a few hours the patient died. On examination, the milk and water were found in the posterior mediastinum! and we have seen a bougie take the same route in overcoming a stricture which was not in existence.

### Malignant Disease in the Œsophagus.

"The discrimination of malignant disease in the œsophagus may, under some circumstances, be doubtful, but where the complaint has existed some time, and the symptoms are considered with care, the seat and cause may almost always be determined with precision. In some few cases, exhibiting every peculiarity of scirrhous structure, the characteristic pains have not been apparent; but the exceptions are extremely rare.

The presence of the peculiar heat and darting pains of scirrhus, may be held fully to establish the existence of this disease, while their absence only leaves the question doubtful. The discrimination of soft cancer, or fungoide tumour, may be assisted by recollecting, that the constitutional tendency in this disease is always to irritation; a condition, the presence of which will always render its existence probable; and, if seated in the upper part of the œsophagus or pharynx, there may even be the peculiar darting pains along the eustachian tubes observed in scirrhus." 97.

We confess that we have witnessed cases of malignant disease in the œsophagus, more particularly of the fungoid kind, unattended with pain, or with any other symptom than difficulty of swallowing. In one or two of these cases their real nature has been dubious, and the diagnosis mainly hinged on the peculiar aspect of the patient. The following case is not uninstructive.

Case.—*Stricture from Fungoid Tumour in the Pharynx.* Mrs. H., 36, had been some years subject to aching pain and difficulty in swallowing, whenever she took cold, a complaint first felt in a severe sore throat. The small silver ball would not pass an obstruction at the lower part of the pharynx, from whence a ropy mucus was freely excreted. The throat exhibited no appearance of disease, but externally, the right side, opposite the larynx, felt rather full, soft, and tender to the touch. Although the obstruction in the œsophagus was spasm only, the instrument often introduced, during several months, could not by gentle pressure be made to pass beyond it. The pulse, weak and small, was liable to high excitement from the slightest cause. The local distress was a difficulty in swallowing, and in passing flatulence ; occasionally the throat felt heated and painful, shooting laterally to the ears.

The stricture not relieved, the instrument was laid aside. Once, at her urgent request, introduced, it passed only to the contraction, and brought up some minute fragments of a soft solid substance, as if from the ulcerated cavity of a medullary disease. She continued to sink, and, after five months' suffering, died.

*Post mortem.*—Mr. Howship found the cellular tissue at the posterior part of the pharynx infiltrated with a pulpy white matter, as in soft cancer : this deposit at the right side of the pharynx having formed a tumour, and ulcerated through the mucous membrane ; and into this cavity the instrument had passed on the occasion of its manifesting the characters of the disease.

A longitudinal section down the posterior line of the pharynx demonstrated the muscular structure, converted into a brownish, semitransparent, soft substance. The mucous membrane was extensively ulcerated nearly round the cavity, and almost the whole depth of the pharynx. The stricture, still contracted, was somewhat thickened ; below this point the œsophagus was healthy.

We have seen a very similar case, and, in that, the instrument passed into a cavity in the fungoid mass. How careful, then, we should be with instruments ! The gentlest manipulation, the most cautious judgment, are requisite in treating these cases.

### ABSCESS IN THE ŒSOPHAGUS.

The formation of matter, says Mr. H., between the coats or in the cellular tissue of the œsophagus, is, in common, easily discriminated. The probability of such an event may sometimes be inferred from preceding injury, although accidental cold or constitutional predisposition may induce it.

This event is announced by febrile excitement, local sense of heat, throbbing, and tumor, with pain and difficulty in swallowing. The sudden rejection of purulent matter may demonstrate the fact, but the course and event of the case will be still uncertain, dependent on the seat, extent, connexion, and tendency of the abscess, and no less upon the manner in which it has opened ; points that cannot always be determined with precision.

We confess that we have seen cases of suppuration between the coats of the œsophagus by no means so easy of discrimination as this would lead us to imagine. The difference consists, we apprehend, in the fact of the suppuration being circumscribed or diffused. If the former, diagnosis may be easy—if the latter, obscure. We may cite an instance of the former.

*Case of Abscess in the Œsophagus.*—S. N., 28, with severe cold, felt swelling in the throat, just opposite the top of the sternum, acutely painful, especially in speaking. Of this she got better, and nearly recovered, but the symptoms soon returned with increased violence. She was awoke in the night, nearly suffocated with an acutely painful swelling situated just behind the root of the trachea, which, in the attempt to swallow, felt as if the part was turned over. The spot in front of the trachea, she said, was extremely tender to the touch. Easy in that position, she was obliged to remain sitting up through the night, with great anxiety, but little fever. In the morning, endeavouring to swallow a little tea, it went down to the swelling, but no further; and with cough and violent straining it suddenly returned, together with a considerable quantity of yellow thick pus. She felt instant and great relief, from pain, swelling, and dyspnœa, rapidly improved, and soon recovered.

Mr. Howship relates no case of diffused suppuration. The symptoms, such as we have seen them, have been rigors, pyrexia, uneasiness about the throat, anxiety, low fever and delirium, death.

We may introduce another case of circumscribed abscess and ulceration of the œsophagus, related by Mr. Howship, and ending fatally.

*Case.*—Miss L., 62, under care of Mr. Carrick, had uneasiness at stomach, some difficulty in swallowing, and occasional regurgitation of food, from supposed dyspepsia. Solids, in small morsels, went down slowly, exciting a dull, heavy pain, extending from the pit of the stomach to the back bone; otherwise in health. The appetite was good, yet nearly every thing returned, sometimes with a ropy mucus. Nutritive clysters ordered, when combined with an opiate, soothed and allayed the feelings. For some weeks, as much as several pints of glairy mucus was rejected daily; stools clay-coloured, and destitute of bile. Injections of gruel came away in an hour, but those of mutton broth remained quiet altogether.

In about a month she felt something give way, apparently where the tender and painful obstruction had existed, and pus, streaked with blood, was rejected by the mouth. She could now swallow, bowels acting, and stools tinged with bile. Instead of a quart of mucus daily, she now spit only a little pus, but complained of occasional flushes of heat. About a fortnight after this, she gradually declined, sunk, and died.

*Post-mortem.*—In the right chest we found the healthy and partially adherent lung floating in nearly eight ounces of fetid pus, which had issued apparently from an ulcerated opening; the œsophagus, right lung, and stomach, were removed. Laying open the œsophagus, it appeared that about three inches above the cardia it was ulcerated. The adhesion between the lung and the diseased œsophagus was ulcerated through, from the cavity of the abscess between its coats, passing its contents outwards into the chest, and inwards into the œsophagus, where the ulceration of the mucous lining, and exposure of the cavity of the abscess, was more extensive. It appeared that inflammation of the cellular tissue of the œsophagus had induced extensive effusion of albuminous and purulent matter between its coats, with subsequent ulceration of the mucous membrane, allowing the escape of pus, and exposing the albuminous matter involved in the cellular texture, as found on examination.

### ANEURYSM.

A considerable number of cases of aneurysm are reported. We are induced to quote the following, because it may inspire a wholesome caution.

*Case of Aneurysm of the Arch of the Aorta, terminating fatally in a Warm Bath.*—Mrs. S., aged 46, complained of constant oppression and uneasiness, with occasional sharp pains shooting through the back bone, down the left arm, and up that side of the neck. Gentle exercise induced increased difficulty, pain, and cold perspiration. The same symptoms sometimes came on in sleep, with frightful dreams, obliging her to start up, and sit for some time supported with pillows. Pulse 80, soft and weak. In the palpitations, the action of the heart sometimes intermitting, would cease to vibrate, then flutter quickly, and so gradually recover. The pain at the heart, passing into the neck and arm, was occasionally so severe, that the arm for a time lost all power and feeling. Finding herself, as she said, sufficiently weak, she would neither submit to lose blood nor restrict her diet, but begged to be allowed to visit Brighton. When there, she was induced to get into a warm-bath, but was no sooner in than she entreated to be taken out again, and instantly expired; in all probability from the sudden relaxation of the vascular system inducing rupture of the sac, and fatal hæmorrhage.

We think that it was the acceleration of the circulation that gave rise to this. The warm-bath is far too indiscriminately used, and when any disease of the heart or arteries exists, it ought to be refrained from. In febrile disorders, attended with much vascular action, the warm-bath is equally dangerous. Such is the case with rheumatic fever, and metastasis to the heart has been the consequence of the employment of the warm-bath. The case before us is a warning, and as such we address it to our readers.

*Scirrhous Pylorus mistaken for Aneurysm of the Abdominal Aorta.*—This case we introduce because we have witnessed a similar one.

*Case.*—J. B., 45, under care of Mr. Hora, had received a blow upon the chest, twenty months before, and had never felt well since. As he lay on his back in bed, a pulsation was so evident, that the hand laid on the scrobiculus cordis, perceived the action of the heart, over the aorta, beating powerfully. Pressing the tumor, the size of a small orange, laterally, it still seemed to expand, or pulsate, in that direction as well as forward. The patient, however, was not, as he should have been, requested to place himself on his hands and knees, a position that would most probably have cleared up the doubt, by rendering the tumor at once pendent, and independent. Several medical gentlemen who saw the case, agreed in the opinion that it was aneurysm.

The tumor, several months increasing, had first attracted notice by its painful throbbing. When painful, it generally disturbed his stomach with sickness. He soon died.

*Post-mortem.*—On examination, the tumor, which latterly had much increased, proved to be disease at the pyloric end of the stomach. A scirrhous induration, which, with a considerable adipose deposit, had drawn down the inferior end of the stomach, so as to place the seat of disease in front of the spine, and immediately over the aorta.

During the past Winter we saw, with Mr. Barrett Marshall of Chelsea, an elderly man affected with a pulsating tumor in the belly. It was about the size of a small orange, though flatter and more oblong. It was situated in the hypogastric region, though moveable to either side. It pulsated strongly. When diverted to one side, the pulsation was less distinct. It was conceived to be aneurysm of the aorta or of one of its leading branches. We were of opinion that it was either malignant tumor in the omentum or scirrhus of the pylorus. The ordinary symptoms of the latter were absent. The man died, and scirrhus of the pylorus it proved to be.

### Inflammation of the Veins.

Mr. Howship relates several cases of obstruction of the venous system and of inflammation in it. We shall pick out some of the more instructive.

*Case of Inflammation of the Posterior Tibial Veins, on the Decline of Pneumonic Inflammation.*—Mr. G., 32, under care of Mr. Love, was convalescent from an attack of inflammation in the lungs, when he complained of acute pain in the leg, in the course of the large vessels downward from the popliteal space behind the tibia, with total incapacity to stand upon the limb. The pulse was quick, skin hot, and tongue white, with thirst. Mr. Howship found no external heat or tenderness, only an increase in the deep-seated pain on compressing the gastrocnemii.

The mercurial influence, promptly established by the blue pill with opium, aided by repeated leechings, fomentations, and abstinence, soon relieved and removed this complaint; so that, in three weeks enabled again to place his leg to the ground without aggravation of the pain, in six weeks he was quite well.

We notice this case, because we believe it is not very rare. After operations, injuries, inflammatory affections attended with the formation of pus, secondary inflammation of the posterior tibial veins, and, if we mistake not, the sural veins, is not uncommon. The calf of the leg swells, is slightly œdematous yet tense, a faint flush may suffuse it, there is great tenderness on pressure, and much constitutional disturbance. Sometimes the patient dies, sometimes he recovers—in all instances the affection is severe, and should be understood.

*Case of Varicose Veins attacked with Gouty Inflammation.*—J. P., 47, a postman, had been long subject to varicose veins and cramps in the left leg, where there was a cluster of very large varices, in some parts filled with coagula. All his life liable to uneasy and relaxed bowels. A troublesome diarrhœa checked by carminative and alkaline medicines, he the next day complained of pain in the left foot and ball of the great toe, with heat, redness, and swelling; the pain shooting violently up and down.

In a few days the medicine had much relieved the relaxation in the bowels and inflammation on the foot, but at the same time the cluster of varicose veins, above and below the knee, became excessively heated, inflamed, and painful, neither enduring the least pressure nor the slightest touch. It was clear the gouty action, suddenly shifting from the foot to the leg, had as suddenly seized upon the enlarged veins. In three or four days the affection shifted back, his foot again becoming tender, painful, and inflamed, and the veins on the leg in the same degree easier, and able to bear examination and pressure as well as ever.

Mr. Howship passes to Diseases of the Liver. Among his cases we find one of

*Inflamed Liver, with Obstructed and Punctured Gall-Bladder.*

*Case.* A. N., 34, at the age of thirteen, had loss of appetite, headache, fever, and jaundice; in about a month he recovered. At thirty, in India, attacked with pain in the right side and shoulder, with sense of internal swelling, he was again relieved by treatment. In three or four years, his health reduced and his legs swelled and ulcerated, invalided and sent home, he was taken into St. George's Infirmary, still sensible of internal swelling, which now manifested itself outwardly beneath the cartilages of the right ribs, with pale stools, and a bilious tinge upon the skin. Fomentations applied, a central softness induced Mr. Heaviside to puncture it, and about two ounces of a thin purulent bilious serum flowed out. For many weeks, pure bile alone was discharged. Several ounces flowed off daily. In three months, the discharge became scanty and purulent, and the opening contracted and healed, the stools re-assuming their healthy bilious colour, and the patient his feelings of perfect health.

We believe this is not the only case that has occurred of a distended gall-bladder being taken for hepatic abscess and punctured. And it has not happened, in every instance, that the bile has been purulent, or that the patient has recovered.

*Permanent Spasm of the Gall-Ducts, occasioning Death.*—A. B., 18, died after forty-eight hours of excruciating pain just in the seat of the gall-bladder, shooting through to the back, positively relieved while blood was flowing, but only transitorily. On examination after death, Mr. Howship "found a slight adhesion of the anterior edge of the lower right lung, to the diaphragm. In the abdomen the small and large intestines contained a white mucous matter, but no trace of bile. The liver perfectly healthy, the gall-bladder large, tense, and full. Stomach inflated, soft, pulpy, and discoloured, with spots of blood effused into the cellular tissue of the villous lining. The duodenum, gall-bladder, ducts, and part of the liver were removed for subsequent examination.

The gall-bladder opened, a ropy greenish black bile flowed slowly out, a little of which, rubbed in a bason of water, gave a diluted tinge of a brilliant warm yellow. A probe lightly introduced would not pass, nor could I at all find the opening from the gall-bladder by the probe. The ductus communis punctured with a lancet about its middle, a very small probe, with some difficulty, passed through it into the bowel. In the opposite direction it passed with tolerable ease by the hepatic duct into the liver, and also with difficulty into the entrance of the ductus cysticus, being stopped half an inch short of its termination in the gall-bladder. I attempted, in vain, to pass the smallest blunt wire, the duct being apparently completely impervious. Carefully dissecting off the peritoneal coat, I found the impervious duct, solid, contracted, tough, and opake, like a soft ligamentous structure; the open part of the biliary passages having all the characters of health.

The ducts, placed on a piece of glass, in a cool place, I laid aside and examined six hours after, and found, that air, by a blowpipe, now passed through that portion of the duct I had concluded obliterated; and when further dissected for putting up to demonstrate the contraction, it had become as perfectly relaxed as any other part of the tube.

It was thus unexpectedly demonstrated, that the whole obstruction had been spasm, and it was clear that the most complete contraction detected in one part of the duct had probably existed in another also, and therefore, that the ductus communis, during life, had been also perfectly closed by spasm, preventing the bile from flowing into the bowel."

*Convulsive Cough from Biliary Calculi.—Case.*—Mrs. C., 53, was for thirty years subject to hard dry cough, which no medicine would relieve, occasional paroxysms of most extreme pain at the stomach, sickness and vomiting, depressed pulse, and cold sweats. The stomach rejecting every thing, during the attacks, in the last of which, of unusual severity, she sunk and died.

*Post mortem.*—There were slight adhesions between the fundus of the gall-bladder, omentum, margin of the liver and diaphragm. The gall-bladder opened, contained healthy bile, and a quantity of small angular gall-stones, three drachms in weight. The other viscera sound: it appeared that these concretions were the only existing cause of irritation.

### Spasm of the Stomach.

Mr. Howship thus describes the causes of this, and illustrates his observations by cases.

"Spasm of stomach, from the irritation of biliary calculi, has been shown to induce most intense pain, readily distinguished by the sudden accession and decline of the attack, the rejection of everything taken, the pain and tenderness at stomach, the slow pulse, faintness, and cold sweats, by the sudden relief afforded by antispasmodic medicines, and by the condition of this organ thus affected, being occasionally found after death to be a close contraction, not of the central part alone, but the whole pyloric portion of the stomach; the cardiac extremity, although relaxed, being from irritation deprived of its power of retention.

Painful spasm of stomach, exhausting the powers of life, and apparently induced by mental distress, either alone or assisted by gouty influence, may be regarded as a consequence of extreme irritation, precisely similar to the above, to be discriminated to a certain extent by the same train of symptoms.

A contraction less in extent, but equal in degree, presents itself to notice in the exceedingly rare case of central contraction of the stomach, in which it may be presumed that the inconvenience sustained, scarcely going beyond diminished power of retention, depend on the small extent of contraction, and its not being liable to fits of irritation. The probable existence of this condition, derived, as I have seen it, from hepatic irritation of moderate intensity, may be suspected from frequent uneasiness, but little pain at stomach, by retention of the smallest portions of the mildest nutriment only, and by the evidence of hepatic disease, pain, tenderness, or tumor, about the liver, perhaps with jaundice.

The last form of pure irritation to be noticed, operating to destroy life, by weakening the powers of the stomach, may occur, as in one instance, from the confinement of the smallest bit of omentum in an almost imperceptible herniary protrusion; a case, the discrimination of which, notwithstanding its obscurity, would only require attention to the history and to the state of the part." 207.

*Purulent Effusion between the Coats of the Stomach.*—We introduce this case because we once witnessed, at St. George's Hospital, a like one. We never saw but the one, and we are therefore inclined to think the affection rare. In the instance that fell under our observation, as in this, the patient was a spirit drinker.

*Case.*—Mr. B., 45, an intemperate spirit-drinker, was subject to attacks of pain and sickness at his stomach for fifteen years. The attack was generally preceded, for a day or two, by a distressing, burning heat in the stomach : sickness and vomiting then came on, with outward tenderness, and went off in a few days. These complaints increased in severity; and on the day of his last seizure the severity of pain prevented his taking any food. He felt cold, and had repeated slight rigours, and said he feared he should have spasms at the stomach, which he had heard were dreadfully painful. He soon after felt sick, and the next day, with Dr. Ager, Mr. Howship visited him. Pulse weak and small, 120, skin hot, tongue white, great pain and tenderness at the stomach. Mild saline and diaphoretic medicines, with a blister, were directed. The following morning, the medicines rejected, he became comatose, was slightly convulsed, so remained, and in the evening expired.

*Post mortem.*—On opening the abdomen, the stomach was found not to be discoloured externally, appearing and feeling thicker than natural; it was removed and laid open along the lesser curvature, where the thickening was greatest. In making this and other sections, it appeared that pus, fibrine, and serum, had been freely effused into the sub-mucous cellular tissue through all parts of the stomach. In some parts the effused matter was principally purulent, in others fibrinous, with here and there traces of fine capillary arteries shooting into the newly deposited fibrine. Behind the longitudinal rugæ, the infiltration was a transparent serum. At the lesser curvature, the thickness of the coats of the stomach was nearly half an inch, in consequence of these changes.

*Spasmodic Stricture of the Cardia cured by Blue Pill and Opium.*

"I was once called to visit a gentleman who had a very narrow escape with his life, from spasm closing the lower end of the œsophagus, a circumstance I never witnessed before or since. Neither solid nor fluid would pass; it went down to the spot, remained as a weight, and soon returned. The sudden accession almost proved it spasm, and perceiving that an eruption on the hands, having the characters of rhagades, had receded, it struck me, at the moment, that the pil. hydrarg. would reach the spot, and might possibly relieve the affection in time to save life. I ordered pil. hydr. gr. iij. opii. gr. ss. secunda quaque horâ, which was regularly taken. Within two days the mouth affected, the stricture relaxed, and the patient recovered." 221.

We quite agree with Mr. Howship in the following attempt at *discriminating the symptoms of scirrhus and soft cancer,* in the stomach and alimentary canal.

When, he says, the specific disease is of modified character, inclining to the condition of soft cancer, while the total loss of power, as to function, still assists discrimination, there is little or nothing of that peculiar distress and pain that distinguish scirrhus. An irritable and relaxed state of bowels, is, perhaps, more positively characteristic of the medullary than of the scirrhous form of cancer.

### Spasm of the Colon and Sphincter Ani.

Mr. Howship lays down some diagnostic symptoms of the varieties of spasm that affect the large intestine.

*Acute Inflammatory Spasm.*—Bowels confined, stomach irritable, sudden accessions of violent pain, extreme tenderness of abdomen, with constitutional disturbance, equal to that of peritonitis.

*Chronic Inflammatory Spasm.*—In one instance, originating in a blow, and augmented by an attack of fever, more or less constant pain in the seat of stricture, aggravated by costiveness or exercise, and capable of perfect, though not permanent relief, by blistering.

*Permanent Spasm.*—Habitual confinement, obstinate costiveness, or sudden ileus, and death. Uneasy flatulence behind, but no uneasiness in the stricture. Marked sympathy with the state of mind. Vexation or trouble, suddenly arresting digestion, inducing acidity, flatulence, sudden and excessive mental depression, or dread of insanity.

Occasionally a peculiar disturbance is manifested through the sympathetic system, paroxysms of violent excitement in the action of the heart, with distress and difficulty in respiration coming on in sleep. Loss of retentive power in the stomach, with tenderness of abdomen and prostration of strength. Slightly painful spasms in the part now and then, at first induced on injecting the bowel.

The stricture, in rare cases, the seat of frequent uneasiness, under mental fatigue, anxiety, or confinement of the body to one position, becoming acute, or very acute and capable of relief only by relaxing the mind, or by taking bodily exercise. Enlargement and accumulation behind the stricture, in some instances exciting chronic diarrhœa. In the appearances after death, the occasional decline of circulation and deficient vascularity very remarkable.

*Permanent Spasm of the Sphincter Ani.*—Constant uneasiness, distressing or intolerably severe pain, obtuse or acute, in the sphincter, which, on examination, is found unusually firm, hard, and closely contracted; the pain increased by exercise, or even sitting, and prone to recur with aggravated severity in sleep. Medicine nearly or entirely useless.

Mr. Howship dwells on the advantage of enemata in cases of spasm of the colon. A warm emollient fluid should be introduced with the least possible disturbance to the sphincter, at a temperature of 98°, passed in slowly and gently, waiting if pain arise till uneasiness has ceased, to such amount as may distend the intestine to its fair capacity, and so be retained for fifteen, twenty, or thirty minutes.

He relates two cases of spasm of the sphincter cured by the daily introduction of the bougie.

From among numerous cases of ileus, &c. we select one, for its practical utility, of—

*Intus-susception successfully Treated by Distention of the Bowel.*

*Case.*—Mr. H. was requested to see Miss J. W. æt. four years, seized the preceding evening with pain in the bowels, sickness at stomach, and violent tenesmus, yet voiding only blood. These symptoms still existed with quick hard pulse, hot skin, white tongue, with tense and tender abdomen. Mr. Plumbe had very properly directed leeches and aperients, the latter being rejected by vomiting. An enema also had directly returned. Both considered it as a case of intus-susception of some part of the intestines. Three days passed without relief; abdomen rather less tense, pulse 160, stomach rejecting every thing. It was clear the child could not live, unless something more was done. Mr. Howship suggested the trial of an injection of warm gruel, which, if the intus-susception happened to be situated in the great intestine, might possibly be useful, but could not add to the danger. The fluid was injected gradually, and the action of the sphincter steadily assisted by lateral pressure to prevent the fluid escaping. The great difficulty was its retention, without which, the case was hopeless. As the quantity increased, the abdomen was gently and repeatedly rubbed, and as the operation proceeded, it was observed that although the abdomen was of course more full, it became less painful that before. During the hour occupied in the operation, the skin became cool and moist, the pulse fell to 110, and the perpetual retching entirely ceased. The fluid was then permitted to flow off very slowly, to give the bowels time to contract. The enema brought away some soft fæces. The quantity injected, when returned, was found to exceed two pints. An aperient mixture in small doses at short intervals, previously rejected, now remained quietly on the stomach, soon passed through, progressively cleared the bowels of a large quantity of pulpy fæces, and the child perfectly recovered.

*Abscess between the Rectum and Vagina.*

This is no common case. Its narration may assist diagnosis in others.

*Case.*—M. B. 24, fifteen months previously, from habitual costiveness, had frequent straining to relieve the bowels; and under constant exposure to all weather, drinking spirits, and often sleeping in wet clothes, constant tenesmus, aching at the loins, burning heat, with sickness and retching, came on. The tenesmus, flatulence, and pain in the back, sometimes brought blood with the stools, which generally contained purulent matter. The rectum examined, Mr. Howship found within the sphincter a soft protruding tumor, made up of numerous folds of the inner membrane, through which the finger could find no opening. Notwithstanding occasional relief from anodyne or other medicines, the constant and distressing tenesmus, irritation, and pain continued to increase, until she sunk and died.

*Post-mortem.*—The lower part of the rectum was not contracted, but thickened. The muscular coat, apparently one-eighth of an inch thick, not indurated, yet presenting a semitransparent greyish texture, like incipient scirrhus. Just above the sphincter, the relaxed, abundant, and œdematous mucous membrane, collected into folds, explained the obstruction felt during life.

Between the rectum and vagina, just above the sphincter, an abscess had formed, discharging its contents by an opening into the gut, and continuing to secrete pus from that time until she died. The mucous membrane of the bowel, at various points was ulcerated, from the opening of the abscess to some distance above.

Mr. Howship observes, in reference to this affection, that, "instructed by this and by another case of the certain event, if let alone, finding a young lady so situated, I took the uncertain chance of escape from bleeding, and made a deep section. Healthy action and granulation followed, and she recovered; but I never was more near losing a patient by hæmorrhage than in this case."

### *Peritoneal Inflammation, simulating Psoas Abscess.*

*Case.*—E. S., 17, a servant girl, lifting a weight, strained her loins and felt pain in the back; at first trifling and seldom, at length considerable and constant. In twelve months, a swelling formed in the right groin, receiving an impulse in coughing. It broke, and discharged freely for many months, with frequent severe pains at the loins, especially when the bowels acted. Declining in strength, she took bark. The discharge lessened, pain in the loins increased with throbbing, when the stomach sickening, she felt something break in the bowel, not, she said, low down, but high up, followed by an immediate and copious discharge of pus, per anum. The previous distress and aching in the back became much easier, and the discharge gradually lessened. It gathered again, and six weeks after the first, it broke a second time into the gut, and discharged at once near a pint of matter; she gradually sunk, and in three weeks died.

*Post-mortem.*—In the abdomen, Mr. H. found the lower part of the rectum, the uterus and appendages involved and concealed in a quantity of effused fibrine, having the appearance of a very condensed cellular tissue, filling up the pelvis. The rectum adhering to this effused matter, and repeatedly reflected on itself, had received the contents of the abscess, which had formed in the midst of the mass of fibrine, discharging its contents, at first through the groin, and afterwards by an opening high up, into the rectum; but the parietes of the abscess had so contracted, as latterly to be scarcely capable of containing half an ounce of pus.

We certainly have not selected *every* case or *every* observation of interest in this volume. But we believe that we have presented our readers with the majority; and, unconnected as they are, they still are calculated to afford some useful hints to practical medical men.

